# Analyzing Preceding factors affecting behavioral intention on communicational artificial intelligence as an educational tool

**DOI:** 10.1016/j.heliyon.2024.e25896

**Published:** 2024-02-06

**Authors:** Patrick M. Cortez, Ardvin Kester S. Ong, John Francis T. Diaz, Josephine D. German, Singh Jassel Satwant Singh Jagdeep

**Affiliations:** a School of Industrial Engineering and Engineering Management, Mapúa University, 658 Muralla St., Intramuros, Manila, 1002, Philippines; b E.T. Yuchengo School of Business, Mapúa University, 1191 Pablo Ocampo Sr. Ext., Makati, Metro Manila 1205, Philippines; c Department of Finance and Accounting, Asian Institute of Management, 123 Paseo de Roxas, Legazpi Village, Makati, 1229, Metro Manila, Philippines

**Keywords:** Communicational artificial intelligence, Education, Self-determination theory, Structural equation modeling, Unified theory of acceptance and use of technology

## Abstract

During the pandemic, artificial intelligence was employed and utilized by students around the globe. Students' conduct changed in a variety of ways when schooling returned to regular instruction. This study aimed to analyze the student's behavioral intention and actual academic use of communicational AI (CAI) as an educational tool. This study identified the variables by utilizing an integrated framework based on the Unified Theory of Acceptance and Use of Technology (UTAUT2) and self-determination theory. Through the use of an online survey and Structural Equation Modeling, data from 533 respondents were analyzed. The results showed that perceived relatedness has the most significant effect on the behavioral intention of students in using CAI as an educational tool, followed by perceived autonomy. It showed that students use CAI based on the objective and the possibility of increasing their productivity, rather than any other purpose in the education setting. Among the UTAUT2 domains, only facilitating conditions, habit, and performance expectancy provided a significant direct effect on behavioral intention and an indirect effect on actual academic use. Further implications were presented. Moreover, the methodology and framework of this study could be extended and applied to educational technology-related studies. Lastly, the outcome of this study may be considered in analyzing the behavioral intention of the students as the teaching-learning environment is still continuously expanding and developing.

## 1 Introduction

**Fig. 1:**
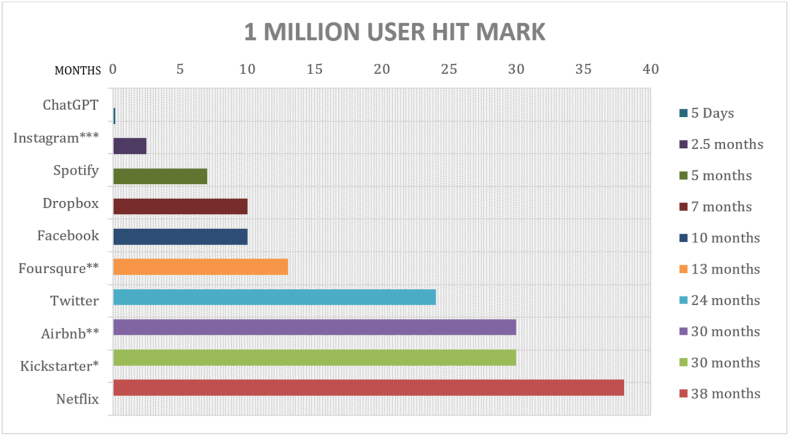
Timeline of Communicational AI for one million users.

Communicational Artificial Intelligence (CAI) has been prominently used as a tool for different purposes in the current generation due to its ability and availability [1]. Different CAIs such as Grammarly, ChatGPT, Quillbot, Jenni AI, Ivy Chatbot, Cognii, Symbolab, Knowji, Otter.AI, and Speechify to name a few, have been used in different activities such as workplaces, education, and personal consumption. In the statistics of Buchholz [2], it has taken far longer for other well-known online CAIs to reach the one million user milestone. Among those closest is Instagram, which reached 1 million subscribers within 2.5 months. Spotify and Dropbox offer an immediate practical utility, also accomplished in five and seven months. However, five days following its November 2018 introduction, the CAI tool ChatGPT reached one million users [2]. Fig. 1 presents the timeline of the different CAIs before reaching the million users obtained from Buchholz [2].

CAIs have been studied in different regions due to their rise and popularity. One of the highly recognized tools nowadays is ChatGPT. Among other CAIs, ChatGPT is used in analyzing possible usage for assisted education. However, it was analyzed only for use during medical licensing exams as a tool for review [3]. Dowling and Lucey [4] used ChatGPT in assisting finance research for idea generation and data identification. The study found that ChatGPT in financial research cannot provide literature synthesis and develop appropriate testing frameworks. Furthermore, ChatGPT offers 43 categories in computer science, marketing, information systems, policy, education, hospitality and tourism, management, nursing, and publishing. However, they also acknowledge the limitations of its capacity [4]. The researchers recognize the ability of ChatGPT to enhance productivity and provide significant knowledge in different fields [5]. With limited studies available, the evident rise and utility of ChatGPT, among others, should be explored due to its potential as a CAI tool especially in the field of education. This became the main motivation of the study since educational technology, its development, and its constant utility worldwide is evident [6]. Moreover, there are a lot of other CAIs being widely utilized, which prompted the need for actual academic use analysis among students.

Another commonly used CAI is Quillbot. It was used to improve the quality of writing and language development among students, similar to Grammarly. It has a significant role for students who create high-quality writing works [7]. It was also used as an alternative paraphraser and reviser for academic works making their outputs look more original [8]. On a different note, Koltovskaia [1] studied the usage and engagement of Grammarly with the students. It assesses and automates written corrective feedback regarding two students' work. However, the accuracy of the feedback was not verified thoroughly due to the different cognitive engagement of the students. Thi and Nikolov [9] assessed the effect of Grammarly feedback on students. Results showed that its feedback was effective and improved the student's writing performance, engaging students to utilize the CAI. Based on the available literature, it could be posited that limited to no studies assessed the use of CAI as an educational tool, specifically in developing countries such as the Philippines. Along with that, no studies were seen focusing on communicational AI use as an academic tool. This presents a significant gap among related studies available. Summarized in Table 1 are the studies found covering CAI.

**Table 1:** Summarized related studies.

Author	Year	CAI Tools	Findings	Limitations
Koltovskaia [1]	2020	Grammarly	They were able to assess the written corrective feedback usage of students using Grammarly.	The accuracy of the feedback was not verified thoroughly due to the different cognitive engagement; only focused on how it is utilized by students.
Kung et al. [3]	2023	ChatGPT	They have concluded that it could help medical students review for needed examinations.	They were only able to identify the use of ChatGPT as a review assistive tool.
Dowling and Lucey [4]	2023	ChatGPT	They analyzed the use of ChatGPT for research, focusing on data generation and identification. It was concluded that theories and frameworks cannot be generated, as well as proper literature reviews are needed.	They were only able to identify the use of ChatGPT among financial research.
Kurniati and Fithriani [7]	2022	Quilbot	They found that Quilbot is utilized mainly for the improvement of student work.	They only focused on one paraphrasing tool, and only identified how it is used among students.
Fitria [8]	2021	Quilbot	They assessed how students utilized this as a way to enhance written outcomes.	They focused only on one paraphrasing tool, and its benefit for students' outcomes and written works.
Thi and Nikolov [9]	2021	Grammarly	Their results showed that the feedback from Grammarly was effective and improved the students' writing performance, engaging students to utilize the CAI.	They only focused on how Grammarly enhanced students' written outcomes, as well as writing performance, and student engagement.
This study	2023	Communicational AI as a whole	This study assessed technology adoption, behavioral, and cognitive aspects of students when using CAIs. The current study provided a holistic approach and findings to highlight how CAI could be used as an educational tool.	This study was only focused on the affecting variables for technology adoption and acceptance, cognitive, and behavioral aspects for benchmarking since no current literature has covered these aspects as a whole.

In order to holistically analyze educational application and technology acceptance, several theories have been made available for behavioral assessment to be possible. One of which is the self-determination theory (SDT). SDT explores how individual variations and societal environments influence various forms of motivation. It is a motivational theory of personality, development, and social processes [10]. Hence, SDT can be a framework used to assess the significance of both the quality and amount of motivation among individuals. It has also provided a unique all-encompassing approach to examining behavior in terms of health, education, and the like [10,11]. Hui and Tsang [12] stated that the key elements of self-determined actions are the ability to act independently and respond to circumstances in a psychologically empowered way. Furthermore, SDT has been used to analyze students' motivation in various academic courses [13]. Krause et al. [14] used SDT to examine an individual's musical participation and well-being. They assessed and compared the potential association of psychological needs to demographic variables and musical activity parameters. With CAIs like ChatGPT, being newly developed artificial intelligence tools, the Unified Theory of Acceptance and Use Technology was employed together with SDT for a holistic measurement of motivation and technology acceptance among consumer behavior [15].

The Extended Unified Theory of Acceptance and Use of Technology (UTAUT2) proposed by Venkatesh et al. [16] attempts to measure variables influencing the individual's behavioral intention for technology usage. It is used as a theoretical basis for determining behavioral intentions in any technological field [17]. Furthermore, the potential of UTAUT2 in identifying the factors that influence the students' behavioral intention toward blended learning is favorable [18]. Mtebe et al. [19] applied UTAUT2, excluding behavior and moderator's effect in examining the educator's usage and adoption of multimedia content in Tanzanian schools. Additionally, Gansser and Reich [20] used UTAUT2 to examine how the acceptance model influences behavioral intention and usage behavior for items including artificial intelligence (AI) in a real-world setting. Ong et al. [21] capitalized on the UTAUT2 solely in measuring the factors that affect the behavioral adaptability of telemedicine applications among Filipino users in the Philippines.

In the context of the Philippines, Filipinos' proficiency in the English language and familiarity with technological advancements substantially contribute to the adept utilization of technology [22]. Therefore, as the main objective of this study, the current article aimed to analyze behavioral intention on CAI as an educational tool in academic settings. Specifically, this study opted to determine the factors using an integrated framework of self-determination theory (SDT) and the Unified Theory of Acceptance and Use of Technology (UTAUT2). The study considered structural equation modeling (SEM) to analyze the factors simultaneously. Furthermore, practical and managerial implications for using CAI in academic settings based on the findings were provided. This is because of the rise of AI utility worldwide for education during the pandemic. As education transitioned back to traditional teaching, there were various effects on the student's behavior. This perspective not only highlights the gap but also shows a problem that needs to be addressed since different perspectives in CAI utility have been both negatively and positively connotated. For example, Coklar et al. [23] explained that there is a negative effect of using these applications among teachers due to techno-stress-related aspects such as orientation, teaching and learning process, and technical issues – especially during the COVID-19 pandemic but was later coped with by faculty members after adopting to the changes [24]. Cotton et al. [25] also highlighted that the rise of these CAI tools could bring about dishonesty among students' academic work such as plagiarism and cheating. Contrary, the study of Melvina et al. [26] presented that other faculty members have seen the benefit of using these CAIs as an educational tool – establishing technology adoption for both students and teachers. Benichou [27] also expressed the heightened utility of CAIs on educational work attainment among students since the development of these applications brought guidance, support, and/or development on students' schoolwork and related tasks.

Stakeholders, instructors, and even administrators are presuming the use of which is viewed as a form of plagiarism or cheating [25]. The study enables the researcher to identify the students' behavioral aspects toward AI as a tool for learning, technology acceptance, and innovation adoption. In addition to the effect, the teachers or professors could understand and identify why students behave differently from the traditional learning tools available compared with available AI learning tools. Lastly, it provides essential benchmark findings for future researchers to recognize the effect of AI as a learning tool. The study can therefore contribute to future researchers using AI to enhance the learning style and innovative learning tools that can be convenient and effective for students and educators.

The structure of the paper unfolds as follows: the initial section encompasses the background, problems, challenges, motivation, research gap, objectives, and significance of the study. Subsequently, the second section delves into a comprehensive literature review and conceptual framework. The third and fourth sections systematically present the methodology and results, respectively. The fifth section is dedicated to the detailed discussion of the obtained results. The theoretical and practical implications, as well as the limitations, were also added, and the last section covered the conclusion of the study.

## 2 Literature review and conceptual framework

**Fig. 2:**
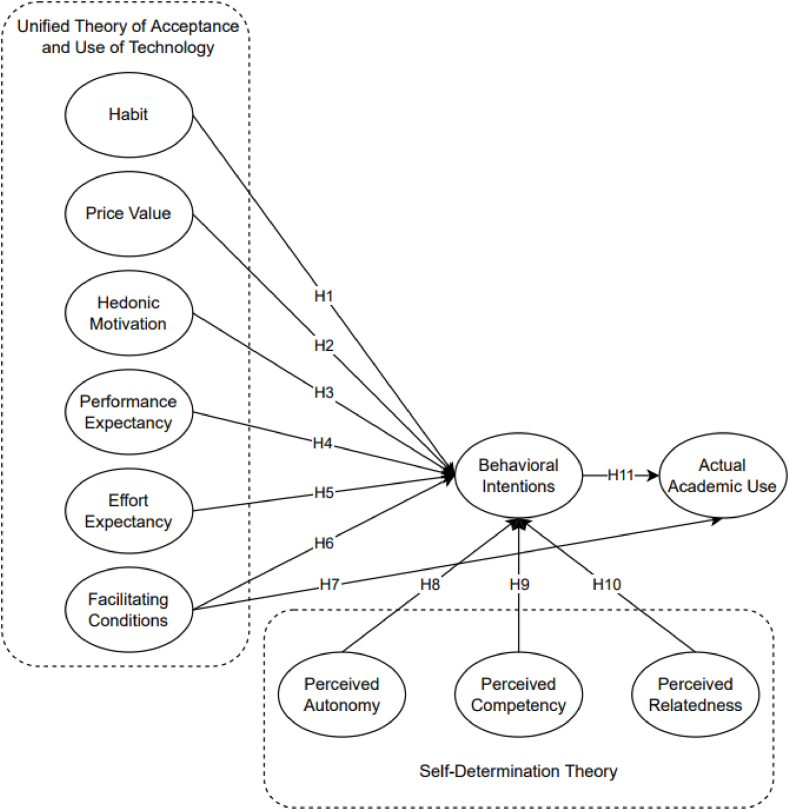
Conceptual framework.

The Unified Theory of Acceptance and Use of Technology (UTAUT2) and the Self-determination theory (SDT) were integrated as the research framework (Fig. 2) considered in this study to holistically investigate the factors influencing the use of communicational Artificial Intelligence (CAI) as an educational tool. Venkatesh et al. [28] only included three moderating factors (age, experience, and gender) and three additional components (price value, habit, and hedonic incentive) to adapt the UTAUT2 model from the UTUAT model to consumer behavior setting on technology adoption [29]. Habit (H), Price Value (PV), Hedonic Motivation (HM), Performance Expectancy (PE), Effort Expectancy (EE), and Facilitating Conditions (FC) are factors that fall in the category. Furthermore, the determinants of SDT were found to be significantly related to the features of a technology-enhanced learning environment for academics and non-academics [30] which was adopted in this study.

H is considered as the tendency of people to carry out an activity automatically by learning and constant usage [17]. Omar et al. [31] verified the association between H and secondary school teachers' inclinations to utilize mobile technologies. As shown by Nikolopoulou et al. [32], H favorably impacted instructors' actual technology usage. It also affected educators' and students' behavioral intentions to accept and utilize mobile internet for education purposes. Therefore, H serves as both an explanation of daily routines and a key determinant of how engaged users will be with the use of a certain technology [17]. The recent update of UTAUT2 indicated more on the appropriate and accepted model in assessing the acceptance and usage of technology for consumers. Existing literature provides evidence, alongside the result explaining the direct significant relation between H and behavioral intention, and actual use of technology among adults [33]. Thus, it was hypothesized that.

H1 Habit has a positive impact on the behavioral intention to use CAI.

PV is a way to quantify the overall gain from employing a technology [17]. According to Coeira [34], the cost of using digital scribes or applications decreases the load of manual documentation. However, the digital scribes are paid less than the users. Past research has supported the impact of PV on technology adoption, a process that is enriching in and of itself and, as a result, has a good sensation and effect on users [17]. Due to the unique notion that it promotes satisfaction, research also supports the relationship between price or its value and behavioral intention in positively enhancing purposeful behavior and adoption [17]. PV is connected to cost-benefit analysis as explained by Jameel et al. [35]. The impact of PV was indicated to be dependent on the advantages gained by students. If the technologies are perceived to have a more significant advantage than the cost, the student will use them. Thus, PV is a mental balance between technology usage and the amount spent. Moreover, the students accepted e-learning when they realized that the usage of this system was more significant than the cost [35]. Thus, it was hypothesized that.

H2 Price value positively impacts the behavioral intention to use CAI.

HM is related to how students perceive the advantages of online learning [35], which is also a significant determinant of behavioral intention [17]. It also presented how HM or perceived enjoyment is a significant determinant of behavioral intention [33]. It is defined as the fulfillment of using applied technology, which directly influences behavioral intention. When students are enjoying and comfortable in using the technology, they tend to use it continuously [35]. According to Omar et al. [31], HM is a predictor of behavioral intention in the context of mobile learning. In Jordan, Al-Zoubi and Ali [36] reported that students' perceptions of enjoyment substantially influenced their intentions to adopt mobile learning. It was discovered that HM positively predicted educators' and students' behavioral intentions to adopt and use mobile internet and a natural desire to begin behaviors. Macedo [33] indicated that most of the older generation has also adopted technology. Similarly, a study by Ramirez-Correa et al. [37] showed how HM influences older generations to use technology for recreation, especially during the COVID-19 pandemic. In addition, the study of Calvo-Porral and Pesqueira-Sanchez [38] assessed that different generations use technology depending on their purpose, showing HM as one of the key determinants. Thus, it was hypothesized that.

H3 Hedonic motivation has a positive impact on the behavioral intention to use CAI.

The degree to which a user expects that a gadget will assist them in completing a job is known as PE while EE describes the level of comfort included in using the technology [39] and influences students' behavioral intention to use e-learning favorably [40]. According to the study of Alblooshi and Abdul Hamid [40], the relationship between PE and EE on students' behavioral intention to utilize e-learning was still strong and favorable. EE is explained to affect people's attitudes toward technology use [41] and plays a crucial role in technology acceptance [33]. According to the study of Tran et al. [42], initial trust and PE in technology were significant predictors of AI adoption intentions. On the other hand, the research of Macedo [33] stated that a consistent UTAUT assumption and result shows that EE significantly affected intention even though their coefficient weights were smaller. In addition, there is an indirect relation between PE, EE, and FC towards the behavior intention of adults regarding the usage of Information and Communications Technology (ICT). Cao et al. [43] hypothesized that people's attitudes toward the adoption of AI-based recruitment systems in the context of human resource management are influenced by these three factors. Pequeña et al. [44] stated that FC has a strong relativity to technology usage. Van Droogenbroeck and Hove [45] hypothesized that FC has a positive effect on behavioral intention and user technology usage. Therefore, the following were hypothesized.

H4 Performance expectancy has a positive impact on behavioral intention to use CAI.

H5 Effort expectancy positively impacts the behavioral intention to use CAI.

H6 Facilitating conditions positively impact the behavioral intention to use CAI.

H7 Facilitating conditions positively impact the actual use of CAI in academic settings.

Fathali and Okada [30] explained that SDT determinants can notably predict perceived competence as the most influential factor because there are challenges and opportunities for learning technical skills in e-learning environments. This was seen to have one of the most impact latent describing how valuable and straightforward the system affects the learning process. To which, perceived competence is an individual's capacity that leads to the success of their tasks and activities [46]. On the other hand, perceived autonomy with flexible learning, and perceived relatedness with computer-mediated communication and social interaction are high competencies and viewed with alarm, particularly by those who have had limited opportunity to interact with the system [47]. Moreover, perceived relatedness indicates the people's urge to be in touch with other members, while perceived autonomy reflects their desire to self-regulate their activities when utilizing a technology tool, like in technology-related applications [30]. Thus, the following were hypothesized.

H8 Learners' perceived autonomy positively impacts behavioral intention to use CAI.

H9 Learners' perceived competence positively impacts behavioral intention to use CAI.

H10 Learners' perceived relatedness positively impacts behavioral intention to use CAI.

Gatzioufa and Saprikis [48] described performance expectations, effort expectations, social influence, and enabling conditions to be variables that impact the desire to utilize technology including three internal elements influencing usage intention: HM, H, and PV. Attitudes toward using AI, perceived ease of use, subjective norm, and behavioral intention were validated using exploratory factor analysis [49], which predicts usage behavior [28]. In relation to this study, these are factors in relation to perceived relatedness among learners, EE, and perceived autonomy. Perceived autonomy is most pertinent to the current study to understand managers' perceptions of using AI for organizational decision-making [43]. Individuals must be encouraged in their autonomy to choose and in developing their competence toward achievement; thus, demonstrating that school students' behavioral engagement as behavioral intentions to learn online is accounted for by their satisfaction with autonomy, competence, and relatedness in digital environments [50]. On the other hand, Nikou and Economides [51] discovered that competence, autonomy, and relatedness might predict users' behavioral intentions to utilize mobile-based assessment for exams when SDT was paired with the technology acceptance model. Since intention is a sign of motivation, this might be viewed as having a solid drive for learning [50] and a person's preparedness to engage in a particular activity and is an immediate result of behavioral action [41]. Their favorable perceptions of using them influenced students' behavioral intention to utilize ICT tools because the SDT domains are significant predictors of intention to use technology [52]. Thus, it was hypothesized that.

H11 Learners' behavioral intention to use CAI as an educational tool positively impacts actual academic use.

## 3 Methodology

### 3.1 Participants

Google Forms was used to disseminate an online survey through a convenience sampling method using different social media platforms [53]. The respondents were asked for their approval before answering and were given the option to leave the survey anytime. The participants included all educational levels from any school in the Philippines to generalize the acceptance of CAI. They voluntarily answered the questionnaire with their full knowledge. Respondents spent approximately 5–10 min completing the questionnaire, encompassing a total of 63 questions, which also covered demographic information. A total of 533 respondents answered the survey from March 2023 to July 2023 and only a minimum of 400 respondents for generalizability is needed [[[53]], [5][4]]]. Students from different universities, high schools, or elementary schools could voluntarily answer the questionnaire. A test for common method bias was used to remove any singular university answer from the respondents using Harman's Single Factor test. From the collected data, a total variance of 34.56% was obtained which showed no common method bias [55]. Presented in Table 2 are the respondents' demographic profiles. The survey was approved by the University Ethics Committee (FM-RC-23-01-39) prior to distribution. In accordance, this research followed the Philippines Data Privacy Act 2012 – RA 10173. The respondents' consent (and some parental consent) was obtained (FM-RC-23-02-39).

**Table 2:** Respondents’ demographic profile statistics (n = 533).

Characteristics	Category	N	%
Sex	Male	293	54.9%
	Female	240	45.1%
	Total	533	100%
Age	Below 16 years old	2	0.4%
	16–25 years old	520	97.5%
	Above 25 years old	11	2.1%
	Total	533	100%
Area of residence	Urban	393	73.8%
	Rural	85	15.9%
	Suburban	55	10.3
	Total	533	100%
Education level	Elementary	0	0%
	Junior high school	7	1.3%
	Senior high school	32	6%
	Undergraduate	473	88.8%
	Graduate Studies	21	3.9%
	Total	533	100%
Total income/allowance (monthly)	Less than 10,000 PHP	350	65.7%
	10,001–20,000 PHP	121	22.7%
	20,001–30,000 PHP	28	5.2%
	30,001 and above	34	6.4%
	Total	533	100%
Frequency of internet usage per day	Less than 4 h	23	4.3%
	4–8 h	121	22.7%
	8–12 h	211	39.5%
	More than 12 h	178	33.5%
	Total	533	100%
Academic Activities performed using Online tools	Short Essays	478	89.7%
	Math Computations	313	58.8%
	Research Papers	453	85%
	Thesis	359	67.4%
	Case Studies	336	63.1%
	Lab Reports	318	59.7%
	PPT	400	75.1%
	Others		0.4%
“How often will you consider using Communicational Artificial Intelligence (Such as Chatgpt, Quillbot, Grammarly, etc.) as an Educational Tool?”	Never	0	0%
	Occasionally	14	2.6%
	Sometimes	151	28.3%
	Often	192	36.1%
	Always	176	33%
	Total	533	100%

The majority of the respondents were male (54.9%), followed by 45.1% female. Most of the respondents fall within the age range of 16–25 years old (97.5%). Additionally, almost three-fourths (73.8%) from 16 to 25 years of age were from urban areas. Regarding educational attainment, most of the respondents were undergraduates (88.8%). The highest percentage of respondents (65.7%) earn a monthly income or allowance below Php 10,000. With these, 39.5% of the respondents have a frequency of internet usage of 8–12 h a day, followed by respondents (33.5%) who use the internet more than 12 h a day. Lastly, most of the students (89.7%) use online tools for short essays. From the collected data, the cumulative extracted squared loading is 48.542%, the percent variance is 48.54%, and the total variance is 26.698%. As explained by Podsakoff et al. [55], the cut-off is 50%, anything less than that presents no common method bias (CMB). Specifically, Bathaiy et al. [56] explained that 26% is deemed significant to indicate no CMB.

### 3.2 Questionnaire

Furthermore, the conceptual framework (Fig. 2) guided the establishment of the adapted questionnaire utilized in this study to determine and analyze behavioral intention analysis on Communicational Artificial Intelligence as an Educational Tool in Developing Countries. The questionnaire had 14 sections [[[2][8]], [3][3]], [5][7]], [5][8]], [5][9]], [[60]]], including the demographic profile, academic activities, frequency of internet usage and considering using CAI, habit, price value, hedonic motivation, performance expectancy, effort expectancy, facilitating conditions, behavioral intention, perceived autonomy, perceived competency, perceived relatedness, and actual academic use. A total of 55 questions were asked among the respondents as seen in the supplementary files. Moreover, the respondents' demographic information collected information on their sex, age, area of residence, educational level, total income/allowance, frequency of internet usage per day, academic activities, and education tools in academic writing as presented in Table 1. A 5-point Likert scale was used in conducting the study to measure the variables from strongly disagree as 1 to strongly agree as 5. Prior to analysis, the Shapiro-Wilk test was conducted to test acceptable normality content. Presented in Table 3 are the descriptive findings. According to Hair [54], the quotient obtained on skewness and kurtosis should be within ±1.96 to have acceptable data collection for SEM analysis. The highest value obtained is +1.914 and the lowest is −1.683, therefore the data collected could be utilized [[5][4]], [[61]]].

**Table 3:** Shapiro-Wilk analysis.

Indicators	Mean	Skewness	Kurtosis	Shapiro-
	Statistic	Statistic	Statistic	Wilk
H1	3.167	−0.237	−0.623	0.381
H2	2.820	0.125	−0.770	−0.163
H3	3.159	−0.299	−0.838	0.357
H4	3.189	−0.278	−0.640	0.435
H5	3.330	−0.503	−0.527	0.955
PV1	3.322	−0.224	−0.117	1.914
PV2	3.403	−0.536	0.319	−1.683
PV3	3.442	−0.640	0.477	−1.342
PV4	3.489	−0.545	0.562	−0.970
PV5	3.176	−0.025	−0.254	0.098
HM1	3.635	−0.740	0.802	−0.922
HM2	3.627	−0.838	0.744	−1.126
HM3	3.627	−0.675	0.569	−1.186
HM4	3.472	−0.504	0.362	−1.391
HM5	3.966	−1.007	1.308	−0.770
PE1	3.927	−0.907	1.657	−0.548
PE2	3.781	−0.829	0.634	−1.307
PE3	3.871	−0.772	0.874	−0.883
PE4	3.850	−0.832	1.092	−0.762
PE5	3.721	−0.703	0.398	−1.768
EE1	3.837	−0.691	0.849	−0.813
EE2	3.751	−0.655	0.603	−1.087
EE3	3.532	−0.508	0.511	−0.995
EE4	3.721	−0.711	0.713	−0.998
EE5	3.850	−0.705	0.469	−1.503
FC1	3.841	−0.666	0.529	−1.260
FC2	3.854	−0.942	1.263	−0.746
FC3	3.725	−0.962	1.155	−0.832
FC4	3.712	−0.708	0.373	−1.896
FC5	3.674	−0.560	0.508	−1.103
PA1	3.734	−0.804	1.419	−0.567
PA2	3.794	−0.806	1.241	−0.649
PA3	3.725	−0.797	0.940	−0.847
PA4	3.657	−0.725	0.871	−0.833
PA5	3.674	−0.686	0.661	−1.039
PC1	3.343	−0.220	−0.249	0.884
PC2	3.365	−0.278	−0.160	1.742
PC3	3.060	−0.081	−0.614	0.133
PC4	3.609	−0.698	0.416	−1.677
PC5	3.476	−0.475	0.360	−1.320
PR1	3.438	−0.604	0.361	−1.673
PR2	3.326	−0.443	−0.247	1.798
PR3	3.206	−0.518	−0.361	1.433
PR4	3.232	−0.501	−0.358	1.400
PR5	3.459	−0.601	0.451	−1.333
BI1	3.815	−0.986	1.538	−0.641
BI2	3.790	−0.946	1.593	−0.594
BI3	3.760	−0.897	1.169	−0.768
BI4	3.785	−0.805	0.726	−1.110
BI5	3.751	−0.780	0.870	−0.896
AAU1	3.528	−0.617	0.696	−0.886
AAU2	3.438	−0.726	0.527	−1.379
AAU3	3.554	−0.820	0.662	−1.239
AAU4	3.283	−0.379	−0.448	0.847
AAU5	3.416	−0.533	−0.517	1.031

### 3.3 Structural equation modeling

Structural equation modeling (SEM) is commonly used in analyzing social, behavioral, and health sciences [61]. Researchers can quickly set up and accurately examine hypothesized links between theoretical constructs as well as those between the constructs and their observable indicators using SEM [62]. It is commonly used due to its capability to assess and determine causal relationships among latent variables [[5][4]], [[61]]]. According to Dash and Paul [61], covariance-based SEM (CB-SEM) may be utilized with established frameworks using the Analysis of a Moment Structures (AMOS) for the causal relationship analysis of latent variables. With this, several studies in relation to technology acceptance have used the CB-SEM type for holistic measurement of behavioral intentions among users. CB-SEM needs to have a complete theoretical model prior to data analysis. The study suggested a precise quantity of dependent and independent variables used in the model. Additionally, latent variables, measurement models, and the quantity of indicator variables are essential to establish a valid and accurate measure of all constructs [[[60]], [[63]]]. Furthermore, Uzir et al. [64] used CB-SEM to assess customer satisfaction, indicating that the model fitness was examined in the measurement model and the presented hypotheses were tested in the structural model. The current dataset included no missing data, according to the SPSS result. Thus, this study opted to consider the use of CB-SEM using AMOS v24 and SPSS v25 for the determination of factors affecting the acceptance of CAI as an educational tool in the Philippines. In reflection to the study done by Dash and Paul [[61]], AMOS for CB-SEM or SMART PLS for PLS-SEM analyses could provide similar insights on the model output. The AMOS software was considered in this study due to its simplicity and the specific steps were addressed in the results section.

In using CB-SEM, the established framework from the integration of UTAUT2 and SDT was considered. After the model build up, the measure items were then placed on the unobserved variables for measurement. Several thresholds were explained along the results threshold, and not being able to reach the threshold indicates an insignificant effect. Therefore, they were removed. Application of CB-SEM in AMOS prompted the use of maximum likelihood estimation and bias, minimization, standardized estimates, squared multiple correlation, and modification indices to run the whole model. These were adapted from the book on multivariate analysis by Hair [54].

## 4 Results

**Fig. 3:**
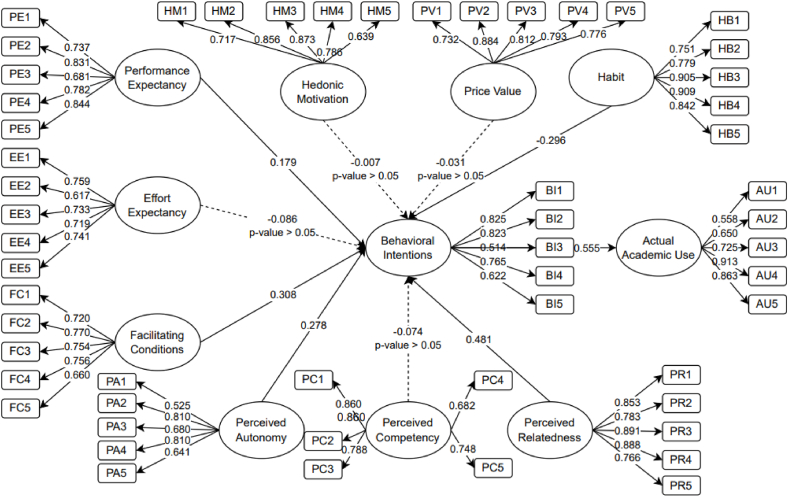
Initial model.

Fig. 3 shows the initial SEM finding for evaluating the student's behavioral intention to use CAI applications as an educational tool. As seen in Fig. 3, performance expectancy, facilitating condition, perceived relatedness, and perceived autonomy have a significant effect on the behavioral intention of the student. Moreover, the insignificant factors can also be seen in Fig. 3 as presented with broken lines (p-value >0.05). These are hedonic motivation, price value, effort expectancy, perceived competency, and facilitating condition to actual use. These might be eliminated, as suggested by Hair [54], to improve the SEM's model fit. The new and final SEM was produced once the non-significant factor was eliminated.

The descriptive statistics for the model's indicators are shown in Table 4. It also shows the initial and final factor loading. Values more than 0.50 are deemed acceptable for these construct variances, which measure the latent of the model. From the output, all measure items were deemed significant. However, the removal of insignificant latent variables due to higher p-value represents no final factor loadings as these were removed to generate the final SEM.

**Table 4:** Indicators statistical analysis.

Variable	Item	Mean	StD	Factor Loading	
				Initial	Final
Habit	**HB1**	3.6763	1.04310	0.751	0.751
	**HB2**	3.4701	1.10044	0.779	0.779
	**HB3**	3.8208	1.07834	0.905	0.905
	**HB4**	3.7669	1.04788	0.909	0.908
	**HB5**	3.8478	1.04066	0.842	0.842
Price Value	**PV1**	3.7611	0.93244	0.732	–
	**PV2**	3.8189	0.91847	0.884	–
	**PV3**	3.7476	0.96055	0.812	–
	**PV4**	3.7707	0.95832	0.793	–
	**PV5**	3.6089	0.98991	0.776	–
Hedonic Motivation	**HM1**	3.7303	0.91136	0.717	–
	**HM2**	3.8613	0.88096	0.856	–
	**HM3**	3.8844	0.87772	0.873	–
	**HM4**	3.7823	0.91786	0.786	–
	**HM5**	4.0193	0.89810	0.639	–
Performance Expectancy	**PE1**	4.0212	0.81346	0.737	0.737
	**PE2**	3.9114	0.90890	0.831	0.831
	**PE3**	3.9268	0.89099	0.681	0.681
	**PE4**	3.9056	0.85013	0.782	0.782
	**PE5**	3.9287	0.91992	0.844	0.845
Effort	**EE1**	3.9345	0.85399	0.759	–
Expectancy	**EE2**	3.8189	0.92892	0.617	–
	**EE3**	3.7803	0.94538	0.733	–
	**EE4**	3.7823	0.92623	0.719	–
	**EE5**	4.0116	0.87867	0.741	–
Facilitating	**FC1**	3.8536	0.86421	0.720	0.720
Conditions	**FC2**	3.8574	0.87814	0.770	0.768
	**FC3**	3.7707	0.91924	0.754	0.757
	**FC4**	3.8651	0.87054	0.756	0.775
	**FC5**	3.8054	0.92722	0.660	0.661
Perceived Autonomy	**PA1**	3.9711	0.81795	0.525	0.525
	**PA2**	4.0520	0.80968	0.810	0.755
	**PA3**	4.0482	0.80754	0.680	0.811
	**PA4**	3.9615	0.82811	0.810	0.809
	**PA5**	3.7996	0.88002	0.641	0.639
Perceived Competence	**PC1**	3.8247	0.96767	0.860	–
	**PC2**	3.8960	0.91574	0.860	–
	**PC3**	3.5896	1.09895	0.788	–
	**PC4**	3.7938	0.93096	0.682	–
	**PC5**	3.7091	0.92485	0.748	–
Perceived Relatedness	**PR1**	3.4143	1.14232	0.853	0.850
	**PR2**	3.7110	1.00255	0.783	0.780
	**PR3**	3.7958	1.04932	0.891	0.891
	**PR4**	3.7707	1.02075	0.888	0.890
	**PR5**	3.8921	0.99221	0.766	0.767
Behavioral Intentions	**BI1**	3.7861	0.89966	0.825	0.820
	**BI2**	3.7765	0.90906	0.823	0.816
	**BI3**	3.1195	1.16463	0.514	0.506
	**BI4**	3.6435	0.97958	0.765	0.755
	**BI5**	3.6994	0.94553	0.622	0.613
Actual Academic Use	**AU1**	3.6763	0.90645	0.558	0.555
	**AU2**	3.8420	1.00293	0.650	0.647
	**AU3**	3.9229	0.94534	0.725	0.722
	**AU4**	3.8497	1.01938	0.913	0.912
	**AU5**	3.8882	0.96013	0.863	0.862

**Fig. 4:**
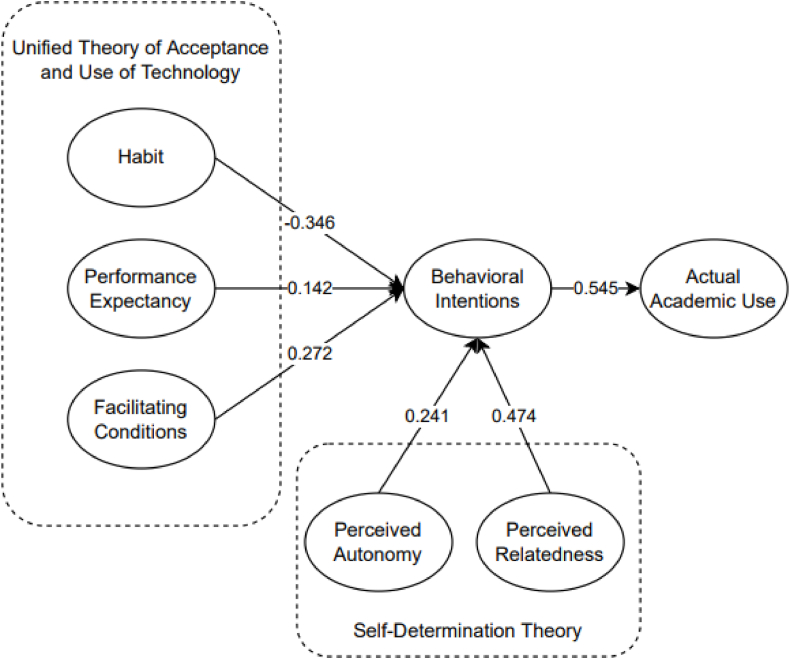
Final model.

Fig. 4 demonstrates the final SEM model of the study. It shows that habit, perceived relatedness, perceived autonomy, facilitating condition, and performance expectancy are significant factors of behavioral intentions of the student, while the behavioral intention of the students was significant to the actual academic use. The most significant factor is perceived relatedness with a result of β = 0.474, followed by facilitating conditions with β = 0.272, and perceived autonomy with β = 0.241. In addition, performance expectancy was considered significant with a β = 0.142. Hence, the behavioral intention of the student was considered highly significant to the actual academic use of the students.

Table 5 presents the composite reliability and validity of the model. In relation to the study of Padilla and Divers [65], the composite dependability for a set of items is measured by the coefficient's alpha. It could be seen that all Cronbach's alpha values are greater than the threshold, 0.7 [54]. In accordance, the composite reliability also presents a high value (≥0.70). Hair [54] explained that both parameters should have a value greater than 0.70 to present an acceptable measure. Moreover, the average variance extracted was also deemed acceptable with values greater than 0.50. Lastly, the test for multicollinearity was performed with the variance inflation factor (VIF). It was indicated that no multicollinearity is seen when the values are less than or equal to 5.0 [[5][4]], [[61]]]; whereas this study only had 3.157 as the highest value.

**Table 5:** Composite reliability.

Factor	Cronbach's α	Average Variance Extracted (AVE)	Composite Reliability (CR)	Variance Inflation Factor (VIF)
Habit	0.922	0.705	0.922	1.774
Performance Expectancy	0.833	0.605	0.884	3.003
Facilitating Conditions	0.851	0.544	0.856	2.826
Perceived Autonomy	0.834	0.513	0.837	3.157
Perceived Relatedness	0.824	0.701	0.921	1.792
Behavioral Intention	0.819	0.508	0.833	3.005
Actual Academic Use	0.858	0.564	0.863	–

Aside from the convergent validity in Table 5, the discriminant validity was also obtained (Table 6 and Table 7). According to Hair [54], the Fornell-Larcker Criterion (FLC) should have diagonal values greatest corresponding to their vertical and horizontal values. In addition, Kline [66] suggested that the heterotrait-monotrait ratio as support for 10.13039/100006600FLC should be performed, which should have a value of less than 0.85. The highest obtained in this study is 0.803. The study of Yang et al. [67] presented similar classifications and indicated that discriminant validity has been achieved.

**Table 6:** Fornell-Larcker Criterion.

	AAU	BI	FC	H	PR	PA	PE
AAU	0.751						
BI	0.704	0.713					
FC	0.555	0.704	0.738				
H	0.515	0.487	0.460	0.840			
PR	0.641	0.585	0.513	0.526	0.837		
PA	0.563	0.702	0.734	0.531	0.594	0.716	
PE	0.583	0.707	0.700	0.607	0.495	0.705	0.778

**Table 7:** Heterotrait-monotrait ratio.

	AAU	BI	FC	H	PR	PA
AAU						
BI	0.762					
FC	0.617	0.803				
H	0.571	0.523	0.505			
PR	0.702	0.614	0.552	0.573		
PA	0.616	0.777	0.798	0.577	0.629	
PE	0.634	0.752	0.763	0.655	0.521	0.79

The model fit indices can be seen in Table 8. Stone [68] stated that fit indices give academics useful information to evaluate how well their structural equation models match the data. In relation to the study, Peugh and Feldon [69] stated that the fit indices in SEM determine if the model is satisfactory in general. Following the suggestion of Gefen et al. [70] and Steiger [71], all model indices from AMOS resulted in an acceptable model.

**Table 8:** Model fit indices.

Goodness of Fit Measures	Parameter Estimates	Minimum Cutoff	Suggested by
Incremental Fit Index (IFI)	0.884	>0.80	Gefen et al. [70]
Tucker Lewis Index (TLI)	0.867	>0.80	Gefen et al. [70]
Comparative Fit Index (CFI)	0.884	>0.80	Gefen et al. [70]
Goodness of Fit Index (GFI)	0.840	>0.80	Gefen et al. [70]
Adjusted Goodness of Fit Index (AGFI)	0.821	>0.80	Gefen et al. [70]
Root Mean Square Error of Approximation (RMSEA)	0.056	<0.07	Steiger [71]

Lastly, Table 9 shows the direct, indirect, and total effects (causal relationship) with their respective p-values. From the results, it could be seen that perceived relatedness and perceived autonomy are the top factors that directly affect behavioral intention, while the same factors have an indirect effect on the actual use of CAI applications as an educational tool.

**Table 9:** Direct, indirect, and total effects.

No	Variable	Direct Effect	P-Value	Indirect Effect	P-Value	Total Effect	P-Value
1	PR → BI	0.474	0.009	–	–	0.474	0.009
2	PA → BI	0.241	0.013	–	–	0.241	0.013
3	HB → BI	−0.346	0.003	–	–	−0.346	0.003
4	PE → BI	0.142	0.049	–	–	0.142	0.049
5	FC → BI	0.272	0.007	–	–	0.272	0.007
6	BI → AU	0.545	0.011	–	–	0.545	0.011
7	PR → AU	–	–	0.258	0.007	0.258	0.007
8	PA → AU	–	–	0.131	0.007	0.131	0.007
9	FC → AU	–	–	0.148	0.007	0.148	0.007
10	PE → AU	–	–	0.077	0.039	0.077	0.039
11	H → AU	–	–	−0.189	0.002	−0.189	0.002

## 5 Discussion

There is an evident rise of CAI tools usage in student performance and student outcomes [72]. Detailed meta-analyses and systematic literature review by Garcia-Martinez et al. [73] showed how CAI (in the current date) has brought better engagement, motivation, and attitude towards learning among students – causing a positive reflection on student academic performance. It was also highlighted how the use of CAI is still underexplored and brings light to the knowledge and application gap of CAI for ethical use in the academic setting. The study of Seo et al. [74] presented how the development of AI brought better and efficient support among students and instructors. Their study, however, was conducted in online classes. Nonetheless, results have presented that these tools could provide prompt, quality and quantity communicational output, support, and an improved connection between faculty and students in learning. The study highlighted the need for assessing responsibility, surveillance, and security issues that may arise from CAI utility. In relation, both the Unified Theory of Acceptance and Use of Technology (UTAUTA2) and Self-Determination Theory (SDT) were integrated to assess behavioral intention among students using CAI applications as an educational tool. This could unfold the knowledge on how students are utilizing CAI in the current educational setting – providing faculty members and stakeholders insights of better CAI tool utility.

Furthermore, structural equation model (SEM) was used in this study to determine the relationship of factors such as Habit (H), Perceived relatedness (PR), Perceived Autonomy (PA), Effort Expectancy (EE), Price Value (PV), Perceived Competency (PC), Hedonic Motivation (HM), Facilitating Condition (FC), Performance Expectancy (PE), and Behavioral Intention (BI) to Actual Usage (AU) of Communicational AI application as an educational tool.

Perceived relatedness is a significant factor in the behavioral intention of the student in using CAI tools (β: 0.474; p = 0.009). The excellent result of the study showed that the students use CAI applications as an educational tool because of peer interactions and influence making them recommend its usage to others with similar academic goals. Through these conversations, several students could use CAI applications allowing them to interact more, feel closer, and empathize with their peers, which was supported by Yoong et al. [72]. From their discussion, it was stated that peer evaluation and feedback empowered and enabled the students to improve [72]. Hence, students' behavioral intention of using CAI applications was based on their intention. In contrast, Filade et al. [73] explained that students do not use CAI based on their peers, showing that using CAI was solely based on their intention. Compared to their study, CAI applications are used based on the goal and potential to improve the student's output. This explains why their findings deemed peers to possess an insignificant effect on the behavioral intentions of using CAI as an educational tool. Among the related studies, Kung et al. [3] provided insights that since medical students, along with their classmates, have proven the use of ChatGPT as an assistive tool expressing a significance on perceived relatedness. The findings of the current study demonstrated that if students would utilize CAI in general as an academic tool, it would be easy for others to adopt this as well [75].

The findings also revealed that students' behavioral intentions when utilizing CAI applications as an academic tool are significantly influenced by their perceptions of autonomy (β: 0.241; p = 0.013). The students feel freedom and can express their opinions when using CAI for their academic work because it suggests improvements rather than changing them automatically. Students also find the suggested option interesting for improving their work. It also suggests that students have control over their academic work while using CAI applications. Lastly, students have freedom of action in learning the course beyond the classroom. They can take their time to learn how to use the CAI applications to maximize its potential. Melvina et al. [26] stated that teachers of English as a foreign language (EFL) expressed favorable opinions about using technology to encourage student autonomy. Most instructors have already employed technology in the classroom to teach languages, including the Internet and other web programs (e.g., Quizlet, Grammarly, English Central, Padlet, and Mentimeter) that can foster student autonomy. However, the actual use of CAI applications has an indirect effect since the main problem of the students was internet connection. Comparing their findings to the study, it is seen that perceived autonomy significantly affects the students' behavioral intention in using CAI applications as educational tools. Both studies show that students openly use CAI applications to improve their academic work. In turn, these behavioral control mechanisms increased more independent types of goals. Additionally, the result from Table 5 shows that perceived autonomy indirectly affects the actuality of the student. Compared to the sole assessment on Grammarly [1,9] and Quilbot [7,8] by related studies, the effectiveness still depends on the users. Since these CAI tools are generative in nature, it is still up to the users to provide the final output. For example, among studies [1,9] Grammarly only provides suggestions, and students are the ones indicating the final sentence for the written output. Thus, this presents that CAIs are used as an assistive tool, rather than a replacement for academic task generation.

According to the study of Hunde et al. [76], facilitating conditions influenced students' behavioral intention to use e-learning. In line with this, the result showed that facilitating conditions are a significant factor in behavioral intention toward using CAI as an educational tool (β: 0.272; p = 0.007). The students have the necessary resources to use CAI since the school is providing some of it for free (e.g., Grammarly). The students also have the knowledge and skills to use CAI since it was introduced for a specific purpose, such as conducting research. They can also easily seek help when they have difficulty using CAI, which allows them to get closer to their peers. However, Bervell et al. [77] stated that the facilitating condition is insignificant to the actual use because students find the platform easy to use. It also suggests that students adapt to gather resources. Comparing their findings to this study, it is seen that students did not find its use difficult; meanwhile, the result of this study explains otherwise. It was also seen that the previous study gathered resources besides CAI applications, making facilitating conditions unimportant.

On the other hand, it can be noted that universities and institutions provide CAI applications for free and even conduct orientation seminars on maximizing its use; this is in contrast with the previous study where students can only access CAI applications if it is free. Thus, this presents how facilitating conditions are one of the highly significant factors affecting behavioral intentions among CAI applications. Contradicting the study of Dowling and Lucey [4] wherein limitations of ChatGPT were seen, the current study presents that students have the capability to utilize other technologies to encompass the limitations of another. It could be posited that since the CAIs are used as assistive tools, they are leaning more on guidance for improvement of academic outputs when needed.

Performance expectancy is also a significant factor in behavioral intention (β: 0.142; p = 0.049). Students find CAI useful in their academic activities. It became their guide for better outputs and increased knowledge. It corrects grammar, punctuation, and proper word usage, allowing the students to learn how to write effectively (i.e., Grammarly). Furthermore, CAI helps students to finish their academic tasks faster (i.e., ChatGPT, Quillbot). For example, Grammarly automatically suggests changes or starting points that the students find convenient. In relation, CAI also helps the students assess their academic output. According to the study by Zuiderwijk et al. [78], performance expectancy is a significant factor in accepting and predicting open data technologies. It showed that the higher the user's expectation to perform well with open data technologies, the higher the behavioral intention to use it. In this study's case, the students use CAI applications to improve their work. Hence, when more students see improvement of their work by the use of CAIs, the higher behavioral intention in using it as an educational tool is indicated. This finding is similar to the specific CAIs considered in related studies [[1], [2], [3], [4], [5], [6], [7], [8], [9]], where positive responses were seen.

CAI is also not an addictive tool that students consistently use. To which, habit was seen to be negatively significant since they use CAI for academic purposes only and not for daily use (β: −0.346; p = 0.003). In relation to perceived competency, it is an insignificant factor since the students do not use CAI for competing or challenging themselves academically. Instead, they use it as a guide in providing a good academic output. It is a supplemental tool for additional learning or knowledge. In relation, Benichou [27] stated that researchers should consider ChatGPT as an extra instrument to create better quality medical research publications. Their study presented how ChatGPT could be an assistive tool rather than a substitute for creating publications. It should be considered as an extra instrument only and not as a primary tool in creating medical research. Compared to the study of Kung et al. [3], it could be posited that this was significant only during the review process of their respondents. However, it could be posited that after the assistance has been made, students would not opt to utilize this in a habitual manner.

Minghao and Wei [79] stated that the behavioral intention of the nurses in using mobile applications was significant to the actual use. This is because using mobile applications could help them with tasks and lessen time consumption. Based on this study's findings, behavioral intention seemed to have the highest significant effect on actual academic use. It was seen that students were interested in using communicational AI as an educational tool. They have access to it, have plans for future purposes, and would recommend it to others. Students highly utilize CAI because it is made fully accessible and available online. It is also considered a user-friendly platform. Since students were given access by their universities and institutions, they can fully capitalize on the availability of CAI applications for their respective academic goals.

The result showed that actual use is also a significant factor in the students' behavioral intention in using CAI applications (β: 0.545; p = 0.011). The students agreed that everyone can learn more by using CAI for academic purposes. Also, students believe that everyone has sufficient internet access and knowledge on how to use CAI for their academic tasks since its development and utility rose during the COVID-19 pandemic [4,5]. During the home learning setup, students used to spend more time accomplishing their academic tasks, leading to the regular use of CAIs [3]. Thus, the related studies justify why behavioral intention posed as the highest contributing factor affecting actual use compared to the indirect effects. In relation to the study of Zuiderwijk et al. [78], the usage of open data technologies was based on the intention of the users due to its benefits. It is also seen that the intention of the users was based on their own perspective and benefit. It could therefore be posited that using CAI often helps in improving outputs.

Chen et al. [49] stated that hedonic motivation negatively affects the student's behavior because the pursuit of hedonism is not associated with the academic setting. In relation to the study, hedonic motivation is insignificant (p-value >0.050) because students only feel happy or joyful after using AI applications for academic purposes. Further, the student who uses CAI because their peers use it becomes an insignificant factor since it is only based on their perspective. In relation, Oliveira et al. [80] stated that price value and hedonic motivation were not significant in mobile payment applications regarding intention in purchasing technologies. It is because the customer's intention in recommending buying technology was based on the social environment and not the intention. In this study, the price value of some of the AI applications (i.e., Grammarly) was not purchasable online since schools are providing it for free– making it insignificant (p-value >0.050). Moreover, effort expectancy became an insignificant factor since students use it in academic outputs (p-value >0.050). The effort and practice of using CAI became a basic tool in correcting mistakes and in making the student's academic output better.

Overall, students use CAI for guidance in improving their work, connecting with peers, and supporting their academic workstyle. It could be deduced that CAI applications are not used in lieu of educational outputs, but rather, as a help or guide. The students may not further develop their knowledge with CAI applications, but it supports in improving their work. Hence, students can adapt to the changes that universities and institutions can employ in their curriculum as a step towards digitalization. It is also not an addictive tool, unlike social media applications that are used regularly; the purpose of CAI applications in education is to help students significantly. Moreover, students are not influenced by their peers to use CAI applications in their work. They use it according to their academic and technological needs. Furthermore, the flexibility and ease of use of CAI applications for students is a great help and guide in educational use since students seek to improve and provide their best work in their academic tasks.

### 5.1 Theoretical implications

The integration of UTAUT2 and SDT in the study helped the researcher to conclude that perceived autonomy and perceived relatedness were the primary variables. Based on the findings, other factors could affect behavioral intention. The other domain (competency) in SDT could be deemed significant – depending on the use and intent of the technology considered. The integration of the two theories could be beneficial in assessing behavior, acceptance, and actual use of technology applications in educational settings. Thus, other researchers could assess and utilize similar integrated theories for the assessment of technology usage in the field of education. It is suggested that researchers could also integrate the Theory of Planned Behavior (TPB) into the framework to identify the motivational factor of the student in using CAI applications as an educational tool. Moreover, the SEM analysis provided accurate results on the causal relationship of the latent variables considered.

### 5.2 Practical contributions and managerial insights

Based on the findings, perceived relatedness and perceived autonomy were the primary variables influencing students' behavioral intentions to utilize communicational AI applications as educational tools. The result of the study showed that they can guide students in their academic tasks. If the students feel happy and can relate with other students when using CAI applications in their academic tasks, their intention to use CAI applications in the future may increase. It is therefore suggested that universities and institutions encourage students to use CAI applications to improve their outputs responsibly. Additionally, universities should encourage students to use either free or institution-provided CAI applications. Therefore, this encourages students to know how to maximize and navigate the available technology. It can also be suggested that developers could enhance and upgrade some of the features, including one where students could establish interactions with others, of CAI applications. It should allow them to communicate and discuss their outputs. Hence, real-time interaction during CAI application usage for educational purposes can be promoted.

Universities and schools can also introduce and promote free CAI applications to students, as well as faculty members. Seminars, webinars, workshops, or lectures should be conducted to teach them how CAI can help in improving academic outputs and tasks. A student's ability to maximize and utilize CAI applications would lead to an increased intention in improving their academic performance. This can also impact their intelligence and boost their confidence in performing academically, as proven by Ouyang et al. [81]. This will eventually encourage students to use CAI applications in the future, which can lead to its integration in respective curriculums. This possible development would help students learn the recent trends and leverage technological advancements into enhancing their academic outputs, as well as applying in real-life scenarios. The result of this study could benefit students, teachers, and schools by empowering students to enhance their academic skills and promote excellent academic performance.

### 5.3 Limitations and future research

With the study's result and background, there were limitations that needed to be highlighted. First, the study focuses only on determining the behavioral intention of the students in using communicational artificial intelligence applications (e.g., ChatGpt, quillbot, etc.) as an educational tool. No evaluation of these CAI tools was considered. By identifying the different behavioral intentions on using it, a better outlook, suggestion, and academic use may be suggested. Furthermore, the researchers only used SEM analysis. Other studies have suggested that the use of machine learning could help develop and prompt higher accuracy results. In accordance, further consideration of analysis such as analyzing academic output, performance, or development with CAI could be assessed by future research. This could help future researchers to compare and identify behavioral intentions through acceptance and usage of CAI tools. Lastly, different methods may be applied to generated data for sensitivity analyses, or even larger models with several mediating factors [82], Bayesian SEM [83], and if whether repeated samples were collected [84]. Even with positive results and acceptable outcomes, it is believed that the generalizability of which may be congruent only with developing countries since the current data was in the said setting. The current study could be a basis or benchmark on the use of CAI as an academic tool. Further elaboration on the actual utility, data from other countries, and holistic comparison is yet to be achieved. This could be an area where future researchers could delve into to provide insights on the overall use of CAI in the field of education.

**Fig. 5:**
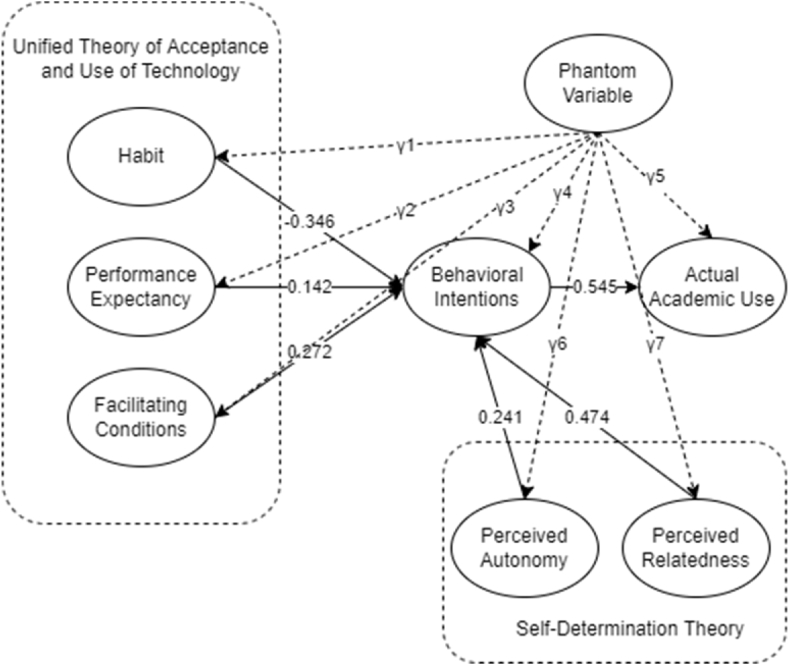
Sensitivity with Phantom variable.$$\sum \begin{array}{ccc} \gamma_{1}^{2}+e_{var1} & & \\ \beta_{1}\left(\gamma_{1}^{2}+e_{var1}\right)+\gamma_{1}\gamma_{2} & \beta_{1}^{2}\left(\gamma_{1}^{2}+e_{var1}\right)+\gamma_{2}^{2}+e_{var2} & \\ \beta_{2}\beta_{1}\left(\gamma_{1}^{2}+e_{var1}\right)+\gamma_{1}\gamma_{2} & \beta_{2}\left[\beta_{1}^{2}\left(\gamma_{1}^{2}+e_{var1}\right)+{\gamma_{2}^{2}}_{+}e_{var2}\right]+\gamma_{2}^{2}\gamma_{3}^{2} & {\beta_{2}}^{2}\left[\beta_{1}^{2}\left(\gamma_{1}^{2}+e_{variable1}\right)+{\gamma_{2}^{2}}_{+}e_{variable2}\right]+\gamma_{3}^{2}+e_{var3} \end{array}$$

Validating the outcome of this study, sensitivity analysis could not be performed in the current study as one of the limitations of using AMOS. It is therefore suggested to future researchers to consider LISREAL or R to run a Phantom Variable. Following the study of Harring et al. [85], it is beneficial for using internal misspecifications for this type of analysis since there were omitted cofounders from the SEM analysis conducted. The reason for which is to reduce bias or endogeneity in causal inferences. As presented in Fig. 5, the model with Phantom Variable is introduced with constant values, γ [[8][5]], [8][6]]]]. Following is the covariance matrix considered, presented in the equation.

Moreover, it is suggested that several metaheuristic approaches may be considered for better assessment of sensitivity [87] or Monte-Carlo simulations [[8][8]], [8][9]]]. Following the study of Naik and Kirvan [90], this could provide better output, even for applications of feature selection.

## 6 Conclusion

Only a few studies were conducted regarding the students' behavioral intention in using communicational artificial intelligence applications as an educational tool. The behavioral intention of the students integrated with the unified theory of acceptance and use of technology (UTAUT2) and self-determination theory (SDT) using SEM analysis has not been tackled. Thus, the knowledge related to the students' behavioral intention in using CAI applications as educational tools was lacking.

The final SEM model showed the different factors, such as perceived autonomy, behavioral intention, and perceived relatedness on actual use, as highly significant factors. The results showed that perceived relatedness has the most significant effect on the behavioral intention of the student in using CAI applications as an educational tool (β: 0.474; p = 0.009), followed by perceived autonomy (β: 0.474; p = 0.009). It showed that students use CAI applications based on their objective and the possibility of increasing their productivity. The students only consider the CAI's suggestions for improvements of their outputs, which explains why their findings deemed peers to possess insignificant effects on the behavioral intentions of using CAI as an educational tool. The students may not further increase their knowledge with CAI applications, but it supports in enhancing their work. Hence, this shows that students can adapt to the changes that universities and institutions can employ in their curriculum as a step towards digitalization – represented by the positive effect on behavioral intention on actual academic use. It is not an addictive tool; the purpose of CAI applications in education is to help students improve significantly. They only use it based on their academic and technological needs. Therefore, proper utility should be taught and practiced in institutions to guide students in maximizing the use of CAI as an emerging technology.

## CRediT authorship contribution statement

**Patrick M. Cortez:** Writing – review & editing, Writing – original draft, Visualization, Validation, Supervision, Software, Resources, Methodology, Investigation, Formal analysis, Data curation, Conceptualization. **Ardvin Kester S. Ong:** Writing – review & editing, Writing – original draft, Visualization, Validation, Supervision, Software, Resources, Project administration, Methodology, Investigation, Funding acquisition, Formal analysis, Data curation, Conceptualization. **John Francis T. Diaz:** Writing – review & editing, Validation, Supervision, Resources, Project administration, Investigation, Funding acquisition, Formal analysis, Data curation, Conceptualization. **Josephine D. German:** Writing – review & editing, Visualization, Validation, Software, Resources, Project administration, Funding acquisition, Formal analysis, Conceptualization. **Singh Jassel Satwant Singh Jagdeep:** Writing – review & editing, Visualization, Validation, Resources, Project administration, Investigation, Funding acquisition.

## Declaration of competing interest

The authors declare that they have no known competing financial interests or personal relationships that could have appeared to influence the work reported in this paper.
