# Supplementary material for: Analyzing Preceding factors affecting behavioral intention on communicational artificial intelligence as an educational tool
Source: Heliyon. 2024 Feb 6;10(3):e25896. doi: 10.1016/j.heliyon.2024.e25896 (PMC10865406; doi:10.1016/j.heliyon.2024.e25896)
Supplement: Multimedia component 1 [file mmc1.docx]

| The construct and measurement items | | | |
| --- | --- | --- | --- |
| Variable | **Code** | **Description** | **References** |
| Habit | HB1 | Using communicational AI applications such as ChatGPT for academic tasks has become a habit for me | Venkatesh et al. [26] |
|  | HB2 | I am addicted to using communicational AI applications for my academic workload | Alam et al. [53] |
|  | HB3 | Using communicational AI applications has been a regular activity for my academic activities. | Venkatesh et al. [26] |
|  | HB4 | Using communicational AI applications has become a natural activity for my academic workload. | Alam et al. [53] |
|  | HB5 | I need communicational AI applications to be able to be satisfied with my academic workload. |  |
| Price Value | PV1 | Communicational AI applications is reasonably priced | Macedo [29] |
|  | PV2 | Communicational AI applications is a good value for the money | Macedo [29] |
|  | PV3 | At the current price, Communicational AI applications provides a good value | Macedo [29] |
|  | PV4 | Communicational AI applications is a good investment |  |
|  | PV5 | Communicational AI applications fits into the budget for students. |  |
| Hedonic Motivation | HM1 | Using Communicational AI applications for academics is fun | Alam et al. [53] |
|  | HM2 | Using Communicational AI applications for academics is enjoyable | Venkatesh et al. [26] |
|  | HM3 | Using Communicational AI applications for academics is entertaining | Alam et al. [53] |
|  | HM4 | Using Communicational AI applications for academics is pleasurable | Alam et al. [53] |
|  | HM5 | Using Communicational AI applications for academics is convenient. |  |
| Performance Expectancy | PE1 | I find Communicational AI applications useful in my academic activities | Alam et al. [53] |
|  | PE2 | Using Communicational AI applications increases my knowledge in academics | Alam et al. [53] |
|  | PE3 | Using Communicational AI applications helps me finish my academic tasks more easily | Venkatesh et al. [26] |
|  | PE4 | Using Communicational AI applications helps me assess my academic outputs | Venkatesh et al. [26] |
|  | PE5 | Using Communicational AI applications improves my academic skills |  |
| Effort Expectancy | EE1 | Learning to use Communicational AI applications for my academic tasks is easy for me | Venkatesh et al. [26] |
|  | EE2 | My interaction with Communicational AI applications for my academic tasks is clear and understandable | Alam et al. [53] |
|  | EE3 | I perform less effort with Communicational AI applications for my academic activities | Venkatesh et al. [26] |
|  | EE4 | I find using the Communicational AI applications for my academic workload proficient. | Alam et al. [53] |
|  | EE5 | It did not take me a long time to understand and use Communicational AI applications as an educational tool. |  |
| Facilitating Conditions | FC1 | I have the necessary resources to use Communicational AI applications for academic purposes | Venkatesh et al. [26] |
|  | FC2 | I have the necessary knowledge and skills to use Communicational AI applications as an educational tool. | Alam et al. [53] |
|  | FC3 | I can get help from others if I have difficulty using Communicational AI applications for my academic tasks | Venkatesh et al. [26] |
|  | FC4 | Communicational AI applications are easy to use even with my mobile phone | Alam et al. [53] |
|  | FC5 | Using Communicational AI applications fits in my academic workstyle |  |
| Behavioral Intention | BI1 | I’m interested in using Communicational AI applications as an educational tool | Prasetyo et al. [54] |
|  | BI2 | I have the access to use Communicational AI applications for my academic tasks and I’m interested in using it |  |
|  | BI3 | I’m planning on using Communicational AI applications for my academic tasks in the future. | Prasetyo et al. [54] |
|  | BI4 | I would recommend using Communicational AI applications as an educational tool to family and friends | Prasetyo et al. [54] |
|  | BI5 | Using Communicational AI applications as an educational tool makes academic tasks interesting | Prasetyo et al. [54] |
| Perceived Autonomy | PA1 | I feel a sense of choice and freedom using Communicational AI applications for my academic activities | Racero et al. [51] |
|  | PA2 | Communicational AI applications in my education provides me interesting options and choices | Racero et al. [51] |
|  | PA3 | I have more control in my academic workload while using Communicational AI applications | Racero et al. [55] |
|  | PA4 | Communicational AI applications gives me more chances to control my academic tasks | Racero et al. [55] |
|  | PA5 | I felt a certain freedom of action in learning the course beyond the classroom using Communicational AI applications |  |
| Perceived Competency | PC1 | I feel that I am better in using Communicational AI applications for educational purposes than other students | Racero et al. [55] |
|  | PC2 | I feel that I have stronger capability and competence than others in academics thanks to Communicational AI applications | Racero et al. [55] |
|  | PC3 | I feel that I am superior to others in academic through using Communicational AI applications | Racero et al. [55] |
|  | PC4 | I have been able to learn an interesting new skill through the use of Communicational AI applications | Racero et al. [55] |
|  | PC5 | I think I am pretty good in academics using Communicational AI applications |  |
| Perceived Relatedness | PR1 | I really like the Communicational AI applications as an educational tool since my classmates are using it | Racero et al. [55] |
|  | PR2 | Communicational AI applications gives me more chances to interact with others | Racero et al. [55] |
|  | PR3 | I feel close to my classmates while using Communicational AI applications for academic purposes | Racero et al. [55] |
|  | PR4 | I have more opportunity to be close to other through Communicational AI applications | Racero et al. [55] |
|  | PR5 | I felt that other classmates had similar goals to mine in using Communicational AI applications for educational purposes |  |
| Actual Academic Use | AAU1 | I think everyone learns more when using Communicational AI applications for academic purposes. | Prasetyo et al. [54] |
|  | AAU2 | I think everyone has sufficient internet access to use Communicational AI applications as an educational tool. | Prasetyo et al. [54] |
|  | AAU3 | I think everyone has the capability to use Communicational AI applications for their academic tasks. | Prasetyo et al. [54] |
|  | AAU4 | I spend a lot of time using Communicational AI applications for my academic workload. | Prasetyo et al. [56] |
|  | AAU5 | I use Communicational AI applications in my academic activities on a regular basis. | Prasetyo et al. [56] |
